# Research Interviews in Historical Practice**

**DOI:** 10.1002/bewi.70000

**Published:** 2025-09-22

**Authors:** Lara Keuck, Soraya de Chadarevian

**Affiliations:** ^1^ History and Philosophy of Medicine Group Department of History Department of Philosophy, Medical School OWL, and Institute for Studies of Science Bielefeld University Bielefeld Germany; ^2^ Department of History and Institute for Society and Genetics University of California Los Angeles Los Angeles CA USA

**Keywords:** historical methods, history of biomedicine, history of molecular biology, interviews, IRB approval, oral history

## Abstract

A key difference between collecting life stories and doing research interviews is the role of the interviewer. While training in oral history may focus on using standard scripts to take a life story, research interviews are motivated by specific questions that arise from particular historical projects and are often not primarily focused on the biography of the interviewee. Therefore, the research interview can be seen as being both less personal with regard to the personal life story of the interviewee and more personal with respect to the foregrounding of the specific interests of the interviewer. Soraya de Chadarevian has been one of the first historians of science to systematically reflect on this and other differences between life story interviews and research interviews. In this contribution, Lara Keuck, who has herself made use of interviews in her research, interrogates de Chadarevian on her approach to research interviews in her historical practice. They discuss how de Chadarevian's personal approach has developed and changed over the past three decades and reflect on the methodological implications that can be distilled from this experience.

## 1 Introducing the Interview on Interviews

A first version of this interview was conducted on May 6, 2024. It started at 6:27 p.m. in Berlin and nine hours earlier in LA, profiting from the digital technology of Zoom. Lara Keuck (LK) and Soraya de Chadarevian (SdC) already knew each other and had informally discussed methodological and historiographical questions of using oral history in the history of science on earlier occasions. For this interview, they wanted to focus on research interviews in SdC's historical practice. They discuss research interviews as a form of oral history that does not focus primarily on documenting comprehensive life stories, but that are directed by the historian's research questions regarding, for example, the interviewee's past experiences, the reconstruction of specific historical contexts and networks, and contemporary interpretations of the past. Keuck sent de Chadarevian some questions beforehand. The interview was recorded and transcribed with the help of artificial intelligence and the human editor Elizabeth Hughes. Subsequently, Keuck and de Chadarevian took turns in editing, correcting, cutting, and expanding the transcript. Above all, they strived to improve intelligibility and readability, for instance, by rearranging some paragraphs and including sub‐sections, but they also addressed feedback gathered at the authors’ workshop in September 2024. They incorporated additional recommendations by two anonymous reviewers in April 2025. The way this interview was compiled, edited, and published reflects a specific understanding of interviewing that is inspired by personal research interests, is skeptical of the special aura of authenticity supposedly residing in the original recording, and that instead views the interviewer and the interviewee as together constructing and co‐authoring the interview.

**LK:** Thank you very much, Soraya, for doing this interview on interviews with me. The idea to use the form of an interview—albeit in an edited version and published as a co‐authored article—was sparked at the workshop that preceded this special issue in June 2023. We were both interested in the question of how generalizable methodological insights from personal experiences with research interviews can be. It seemed apt to discuss this question not in the abstract but against the background of a personal historical practice. I am very happy that you agreed to this experimental form to reflect on what has or has not changed since you started to work with and think about research interviews in the history of science.

## 2 The Personal in Research Interviews

**LK:** My first question refers to the overall topic of the special issue for which we are doing this interview. The special issue is concerned with the difference between life story interviews and research interviews. It is particularly interested in the use of research interviews within the history of science.

Your 1997 book chapter “Using Interviews to Write the History of Science” is one of the first, and one of the most read methodological reflections on this topic.^1^ To start with, can you tell me a bit more about the context of the edited volume, in which your piece was published, and your motivation for contributing this chapter?

**SdC:** The essay appeared in a volume on *The Historiography of Contemporary Science and Technology*, edited by Thomas Söderqvist, then at Roskilde University in Denmark.^2^ The volume built on a conference on that same topic, held at Gothenburg in September 1994, to which I was invited. I had started using interviews for my project on the history of molecular biology and had talked on interviews as a research method before at a small workshop in Maastricht and another one in Cambridge. The conference in Gothenburg was an opportunity to develop my thoughts on the topic a little further.

**LK:** In this chapter from 1997, you already hint at the use of the research interviews in the history of science as some sort of field work. Your chapter reflects on your own use of the interviews when writing a contemporary history of molecular biology. If I compare your paper to other methodological introductions to doing oral history in the history of science, Saul Benison for example, you seem to be less interested in general biographical information about the interviewee.^3^ At the same time, your questions are much more specific with respect to your particular personal research interests. Other interviewers would probably have asked different questions and that made me wonder if it makes sense to say that the research interview is in some respect less and in another respect more personal than a standard life story interview.

**SdC:** Yes, I do think this is a good or a possible way to put it. A research interview, as I tried to define it in the essay, goes less into the full biographical details of the interviewee, and in this respect is less personal.

At the same time, it is more personal with respect to the specific research interests of the interviewer. In some respects, this distinction also maps on the difference between an oral history interview recorded for a multitude of future listeners and researchers, and the interview conducted in the context of a specific research project, as I was pursuing, that is, doing the interviews yourself for your own project.

Of course, there are overlaps between the two genres. Every research interview will include some biographical questions and vice versa, a life story interview will have specific questions on the relevant research area of the interviewee. And when the research project concerns a biography, then of course, also the research interview will be very personal in this first sense, but it will still be more directed than a classic life story interview. The interview technique will probably also be a bit different. Thomas Söderqvist's extended interviews with Niels Jerne come to mind as an example of such a biographical research interview. Söderqvist has also written about what it meant to engage so deeply and for so many hours with one person about whom he wanted to write a biography.^4^

**LK:** Can you tell me a bit more what you mean with different interview techniques?

**SdC:** You see, this goes back—I should mention that before I embarked on my first interview project, I went through the rigorous five‐day residential training course for oral history that was offered by the Wellcome Trust at the time. It was run by Paul Thompson from the University of Essex, a pioneer of the use of oral history interviews in social science research in the UK, and Robert Perks, the curator of the National Life Stories Collection in the British Library Sound Archive. There we learned that in the oral history interview the interviewer should intervene as little as possible, up to the point of becoming a non‐person. This included the way we dressed. We should look and behave as neutrally as possible, ask very open questions, and just let the interviewee speak. I remember distinctly that this was something I struggled with from the beginning. But I think the tension really relates to the difference between this kind of oral history interview and a research interview. In a research interview you intervene much more, you direct the conversation much more. You still want to have these open questions and be open to hearing things that you didn't expect. But you want to gently direct the conversation back towards the questions that you are interested in. When the conversation goes off into another direction, then you let it go off, but eventually you try to steer it back to your questions. Also, who you are, your background, and your research questions—what perhaps today would be called your positionality—come in much stronger in a research interview. I think we may come back to this point in some of the later questions. But this is where I see the difference in interviewing techniques.

**LK:** When did you do that training?

**SdC:** In the early 1990s; 1992 or 1993.

**LK:** And have you ever thought about developing your own training after you had this revelation that that training might have given you some information and maybe also confidence in how to pursue interviewing, while also setting those limitations, such as having to become a non‐person. Or, to put this non‐person issue differently, to mask your personal research interests in interviewing.

**SdC:** When I was in Cambridge, I used to offer a seminar on oral history as part of a methods course for MPhils, and we discussed some of these issues. Actually, I think the article was the basis, but then we would also go into some of the technicalities because the students wanted to do their own interviews. Thus, we would discuss how to prepare for an interview, the technicalities of recording the interview etc. But I think the key question is really how to ask questions and to be aware of the extent to which the questions inform what the interviewee says.

Coming back to the non‐person issue, I think it's helpful for my interviewee to know that I have a background in biology because then they can become technical. Otherwise, they might not go into detail because they think I don't understand it. It's very clear that the interviewee wants to know what you are interested in, because there is much they can tell you, right? They have to decide what to tell you. Thus, I think, telling them a little bit about your project and why you're interested in talking to them seems to be quite necessary to me.

**LK:** Yes, I very much agree. It's actually difficult for me to imagine the epistemic benefits of trying to imagine oneself as a generic historian in interviewing.

**SdC:** I think it comes from the idea of the life story interview that you then record and collect. But I think it's a bit of a fiction. It's based on the idea that the interviewee has this information that can be tapped and it's just about recording that information. But this is not how it works. I don't want to suggest everyone else is naïve. There is this tradition of oral history recording, which is good. It was about giving a voice to people who didn't have a voice and making it part of the historical record. The history from below was a very strong motivation of the oral history movement in Britain and the training was offered with this motivation in mind.^5^

This idea of not being directive with your questions and letting the interviewee speak, this is all fine. I don't think the training for life story interviewing has changed much although I don't know if the Wellcome Trust is still offering these training courses on a regular basis as they used to. In any case, I learned a lot from the training.

## 3 Regulation of Interviews

**LK:** It could be interesting to participate in such a training 30 years later to see what has changed and what has remained the same. But that brings us to the second topic: one other difference between recorded life stories and research interviews is that they are regulated a bit differently.

Increasingly, historians are urged to apply to ethics boards, the Institutional Review Boards, IRBs, before approaching interviewees, and funders often ask for research data, including oral history interviews, to be made publicly available. The research interview seems to have a more liminal status in this respect, so I would be very interested in knowing how your research has been influenced by these developments.

**SdC:** My experience has been a bit different. When I embarked on my first interview project in the 1990s in the UK, there was no need to go to ethical review boards, although consent procedures were in place. That was certainly something that was talked about in the training I received. Things changed when I moved to UCLA. Here, all interviews that are conducted as part of a research project, in history or other social sciences, need to be submitted to IRB review to be certified exempt. I think UCLA is a bit of an outlier here, even in the US. Now, has the fact that I have to go through the IRB process changed my interview practice?^6^ Not fundamentally, I think. The tension between a formal (recorded, with exchange of consent forms etc.) and a more informal interview situation were present before, and both have advantages and disadvantages. I should say that most scientists I talked to were very understanding of these different requirements and willingly signed the IRB approved consent forms. At the end it's a question of trust. Building trust seems to me a very important aspect of a successful interview. IRB vetting can elicit trust, but other factors such as personal or institutional connections, professional credentials, the general attitude you have, all these I think play a role in building trust. We had the situation today: we started talking informally and got straightaway into an interesting discussion. When, then, is the right moment to make it more formal, sign consent forms and turn on the recorder or even the video recorder? It becomes this much more official thing and so the conversation may change, right? But my experience is that scientists, administrators etc. understand this and even appreciate it as the procedure gives it a stamp of professional approval. You can say this project has passed through IRB review, this is a consent form, and it's stamped “IRB approved.” Thus, I think it can work in both ways, depending on the specific situation and the subject matter.

Now there is another aspect. So far, we have talked about the interview process, but your question also points to the increasing demand for making all research materials open access. This is something different with which I have struggled a bit. I certainly agree that if we want to use interviews as historical sources, we need to make them available for reference somehow. If the recordings were clearly treated as research materials and would not be measured against an ideal oral history standard, I think I would be less hesitant making them publicly available. But because of my training I have often felt guilty of intervening too strongly in my interviews.

But of course, if we make them publicly available this changes the consent procedures and potentially makes interviewees more cautious about what they say. It definitely does. I mean, very often an interviewee says, “This is off record,” right? And there is also, I think, a lack of repositories where research interviews, together with other research materials, could be deposited. Thus, I think there is a tension between the need to treat interviews as checkable sources and the feeling that these are private research materials, collected in the context of a particular research project. I was thinking about this regarding my last series of interviews for my book on the history of human chromosome research.^7^ There are collections of interviews for the history of human genetics, even locally at UCLA, but I strongly felt my interviews were of a different kind because they were done with my particular research questions in mind. Thus, I ended up not making them publicly available. But if someone were to ask me about a particular interview from which I quote in my book, then I would make it available, with particular conditions that are consonant with the consent forms my interviewees signed.

**LK:** That's interesting and it brings me back to one question that I had when you were talking about your move to UCLA, because UCLA has, of course, this strong tradition in oral history interviews. There have been many people involved in doing interviews. So, I expect that they were quite aware of interviewing and also had undergone some sort of institutional professionalization regarding consent forms and maybe also IRB approval. Do you know when they started these IRB approvals at UCLA? And do you think that that's specifically connected to the interest and expertise of your former colleagues in oral history and interviewing?

**SdC:** I think you may be referring to the series of interviews on pain research and on human genetics. They were not done in the history department. The project was located in the UCLA Biomedical Library. I think the reason why UCLA has particularly strict rules in this respect may go back to the Moore v Regents of University of California case regarding property rights on bodily parts and consent rules.^8^ The question of course is if history interviews are “human subject research.” The American Historical Association has been pushing back against this and I know that colleagues in other history departments do not go through the IRB procedure. So, there are differences and also some confusion, but the general rule is, if the interview is part of a research project, it must be approved as exempt.

**LK:** In European universities, I see an increasing interest in including IRB approvals now, also in the social sciences and humanities, which wasn't the case for a long time. Many history departments didn't think that it would be important for them to ask for such an approval and that changes now because of the institutionalization of ethics approval and the increased demands to pay attention to data protection laws. I think the effects are quite similar to what you described. There are these aspects of trustworthiness in professionalization.  Funders ask for ethical review procedures and that puts a pressure on the institutions to actually install them. So, I was wondering if UCLA was just quicker in that development.

**SdC:** In cases where you do interviews about some traumatic events–it could be war, persecution or the Holocaust, for example–then you can understand that this might trigger something in the interviewee. These are situations where you want to be very careful and having IRB procedures in place will be really important. And, of course, painful memories or sensitive issues may come up in other contexts as well. I might not have mentioned so far that approval is connected to an extensive online training course on human subject research that is also required and needs to be periodically refreshed.

This is the one aspect. But the strange fact about the IRB approval is that social science projects are always exempt. We know this in advance, but we still have to go through the process to be certified exempt. At least this is the procedure as I understand it and have followed so far.^9^ The process includes going through a very long questionnaire in which 90% of the questions do not apply to our projects. There are questions on research set ups and statistical tools, on drugs and placebos, on side effects, follow up visits, animal experiments, animal welfare. It goes on and on. And even if we know we are exempt, it does not mean we are just waved through. For instance, you have to submit the letter you use to make the first contact with your interviewee, you have to submit the consent form, very important. And this can go back and forth many times. So being exempt doesn't mean that everything is not checked in detail: how to secure the research data is a huge topic, because if it's biomedical data, you have to make sure there is no improper access, no unconsented secondary use and so on. In any case, going through the whole process can take months. That means you have to plan ahead. And you have to keep the approval active. If you don't, the permission to use the interviews you have collected lapses.

I hope if approval will be requested in Germany, they will develop a procedure that fits better with social sciences projects.

**LK:** That is a lot of administrative work that you are describing. Administrative work that I did not do for our interview. Our interview is maybe also a different genre, because we collectively edit and co‐author the publication. Actually, I think it makes me feel more comfortable that way, to remain dialogical all the way down instead of writing about you.

## 4 Changing Practices of Interviewing

**LK:** But back to your way of pursuing and using research interviews: Aside of dealing with those changing regulatory frameworks and demands, or maybe also not so changing, would you say that your practice of research interviews has changed much in the past three decades? Did you develop your own standard practices that have made the process easier? Or have your experiences made you more wary?

**SdC:** That's a good question. I think I have become more assured about this difference between research interviews and other kinds of oral history interviews, because I have been thinking more about everything else that an interview provides besides quotable passages. This is something which I have become more and more interested in. This idea that the research interview is like a historian's fieldwork^10^. And the other thing is that I have come to realize that sometimes our work can be perceived as more threatening than we might expect. I learned this in my recent research project on the history of human genetics. Here some people flatly refused to speak to me about certain aspects of their work. This level of resistance I had not experienced before. Initially I tried to argue that I was not an investigative journalist, that my aim was not to expose anyone or anything. But then I realized that things were not as simple and that there were things that might have seemed okay in the past but are not considered okay anymore. That is a delicate situation, and people just did not want to speak about these things.^11^

**LK:** So, what they considered threatening was related to a specific topic; it was not that they refused to talk to you altogether? They would have an interview with you, but just say that they wouldn't talk about a specific subject or wouldn't reply to specific questions?

**SdC:** No, in the instance I have in mind, I was actually contacting them regarding their work on a particular project and they didn't want to talk to me at all. I think this is not unusual, but I just had not experienced it. And yes, it clearly had to do with the research topic.

**LK:** But did that change your practice? Do you now approach people differently after you had that experience?

**SdC:** Not necessarily, but I was surprised because it was not just one person; it was several people. They had all kinds of excuses why they could not talk to me and I only slowly came to realize that there was an actual issue. I will now be more prepared for something like this to happen.

**LK:** But that means that it was an interesting result in and of itself that people refused to speak with you.

**SdC:** Oh, yeah, this became the story. And, you know, initially I tried various avenues to get to the bottom of the story, but I was met with this wall of resistance and so then my story turned around this impossibility and what that meant. Some well‐meaning people continued to suggest how I could get more information on the matter. But at this point, I didn't even want to find out anything anymore because it would have changed my story. I quite liked the story about not being able to find out certain things and what that meant.

## 5 Epistemic and Social Roles of Research Interviews

**LK:** That nicely brings us to the next question. You've told me before that the research interviews that you pursued for your last book, *Heredity Under the Microscope*, has shaped your research in important ways, although you didn't make much use of verbatim quotes in the book.^12^ Can you elaborate a bit more on the epistemic and maybe also social roles of research interviews in your research practice?

**SdC:** Yeah, this is true. I'm sometimes surprised that I end up using just a few verbatim quotes from the interviews, or very short quotes, considering the number of interviews I do. And so, I think it's a very justified question to ask if it warrants the preparation and the effort and the time that goes into conducting these interviews.

Yet for me, interviews provide so much more than these quotable phrases. They can provide completely new avenues for research; they can create connections that you didn't see before and make you aware of important actors who weren't in your picture. I also use interviews for checking interpretations, for building trust, and for gaining a “feel” for the interviewee.^13^ Knowing your actors, I think, makes a big difference. At the same time, it can of course also make things more difficult.

This maybe is what makes this kind of history so interesting. As historians, I think we're perhaps not so well trained in taking advantage of all the clues we gather when conducting interviews. Or perhaps it has something to do with the way we write. Some people do include more ethnographic descriptions of the interviewing situations as part of their narrative.^14^ These can be very effective. But it is interesting to think more about what else we gain from these interviews and what difference it makes when we know our actors.

It's an aspect that I think is characteristic of writing recent history, whether we talk to our actors or not, as we always know they can talk back and dispute our interpretations of events. Yet with some of our interviewees we talk repeatedly over the course of our research and as a result we get to know them quite well, but of course, all these aspects get lost if we focus on the transcript or when we rely on deposited interviews. I really think it is important to always come back to this distinction as the points we are discussing go beyond the transcript and have to do with the actual encounter.

I've been thinking in terms of metadata and if these metadata should also be submitted with the transcript. You know, every interview includes an extended interaction with the interviewee. There is the first contact. The person responds or doesn't respond, responds enthusiastically, or not enthusiastically. You decide when and where to meet and for how long. You meet in their lab or in their homes or in another place altogether. When you meet them in their homes, the partners may be present. Other times they lead you through their laboratory and show you their last experiments, or they go to their filing cabinets and pick out some stuff for you. They may want to see you again or not; they may still be active in their career or they are retired or have moved on to do other things. All these aspects are part of the interview and help situate what is said. And I do think it is reflected in what we write, even if not in direct quotes.

**LK**: I really like that point about knowing your actors. You already indicated that it also means that they know you then, right? As you said before, you always ask them if you use verbatim quotes. What kind of reactions did you have from interviewees when you either quoted them or maybe also when you had interviews with them, but then you didn't use quotes. You reflect a bit on that also in your 1997 piece, that you're raising expectations and sometimes people will even come to you at a party and tell you: “You're writing this book, you have to interview me!” so how did you find a way to manage these expectations of your actors knowing you?

**SdC:** That is what I meant when I said it can also make things more difficult. It's a delicate balance, because you build up trust and listen to their side of the story. But you also talk to other people, consult written sources and need to balance all the different and sometimes contrasting information you get. This can make it difficult.

But not everyone is super interested in what you do with the interview. Some people are but there is a big gamut. Generally, I find that the people I have spoken to respect the way historians work. They understand that we consult many different sources and that the story they tell us may be different from the story we will tell.

I have found a real appreciation for the footnotes in my texts. We sometimes think we should use footnotes sparingly to render a text more readable. But I have noticed that scientists appreciate the footnotes because they point to the evidence on which the work is based. One scientist reviewer of my last book mentioned this. The footnotes make up nearly a third of the book. That could seem excessive but for the reviewer it proved that everything that was said was well supported. The review also said that the footnotes were a delight to read, which I found amusing.

**LK**: That is very interesting. And it might also be a particular feature of doing a research interview with the scientist, right? Of course, you could do research interviews also with administrators, regulators, technicians or patients. And maybe then that would cause other kinds of reactions, but I can very much imagine a scientist's appreciation of the evidence and the footnotes and that it reflects this understanding of scientific rigor.

## 6 Limitations of Dealing with Oral Sources

**LK:** We've already touched a bit on the next question, which is about the limitations and blind spots of the method. Every method has its limitations and one issue that has been discussed a lot with respect to oral history is the imperfect memory of interviewees and also of their own self‐historicizations that need to be put into relation to other available sources—that are in nice footnotes.

Aside from these general limitations of dealing with oral sources, is there a more specific drawback or caution concerning research interviews that you have experienced?

**SdC:** I think the biggest mistake is to assume that interviewing people gets us to the actual source or the “truth” of what happened. But considering that an interview is a conversation between two people that together produce an account of the past, it is difficult to take this as “the truth.”

The work that is necessary to critically evaluate what has been said, in conjunction with other sources and so on, is what makes interviews such an interesting historical source in my view. With other sources, you can't ask back; in an interview, you can ask back. I think it makes you more aware of the construction of any source. In the interview you become more aware of the construction because you're participating in the process. You're literally witnessing how the source is constructed. You also know very clearly that what you hear is a personal account, an elaboration of something that has happened, filtered through the personal experience and positionality of the interviewee. While written sources have sometimes this aura of being, you know … of course, they are also produced in a particular context and with a particular aim in mind—but they have the authority of the written source. Interviews lack this authority, even if they come from the “original source.” But, of course, you can also bring a document to an interview and say, look, I found this document. Can you tell me a little bit about the context of this document? Which issue was this responding to? Or what did you mean when you wrote that? And so there can be a back and forth between written sources and interviews.

**LK:** I absolutely agree. We've briefly talked about teaching oral history. Students realize while engaging with oral history what is at stake when working with sources in general. To rephrase, it doesn't sound like a limitation or a drawback, but it rather sounds like working with oral sources demands a reflexive mode of the historian.

**SdC:** I agree. I think it's a characteristic of oral history, but I don't think this makes it useless, on the contrary.

**LK:** So, you have nothing bad to say about the method?

**SdC:** It's quite time‐consuming. But you know, it's also just one approach we pursue. It's not an alternative to working in the archives, it's an addition. What is happening to me is that I continue to work on the 1950s, ‘60s and ‘70s, but, of course, time is moving on. And so, even if I keep working on the same time period, there are fewer and fewer people I can talk to. For my current project, which is again on the 1950s–60s, I am nearly out of the time span that allows me to do research interviews.

**LK:** I like those reflections on the practical limitations such as how time‐consuming interviewing is, because I think then the question is: how do you decide whether it's worth the effort to interview someone?

**SdC:** I think you must have questions.

That's also what I say when my students want to do interviews. Just chatting to people will not be very productive. I think you must have questions for it to be helpful.

**LK:** So you have to be at a certain point in your research process where you already have developed some questions in order to say: now it's worth the effort to go through all those approvals and contact the person to actually do a research interview.

**SdC:** I don't know if I want to make this a general rule, but yeah, … I'm just thinking about when I started the last round of interviews … I definitely wanted to know more about certain things—about practical procedures, about the motivations behind certain projects, about how collaborations had started, and I couldn't find the answers in the available records. There were very few records to start with. This is a general problem with recent history as records are often not yet deposited or not yet cataloged. If you do recent history, like from the ’80s and ’90s, the record situation is quite critical. Setting up interviews is a way to get access to papers that people still keep in their filing cabinets (or now on hard disks or the cloud). Some people do interviews mostly with that aim in mind. It's certainly a legitimate practice but I think interviews can provide so much more.

But you have to have questions. Now of course every project is different, but generally speaking I think if you do interviews it's good if you do more than one. And you should not just speak to the top people, but also to the assistants and technicians and the administrative people or whoever was involved. This will provide different perspectives on a story, which is something you can get through the interviews.

**LK:** Okay, so that could be the second thing that is related to methodological blind spots. If you don't think about interviews in a serial manner, you might privilege some perspectives because you did not think beyond the single interview, which is already a time‐consuming thing. Would you say research interviews are not just about interviewing *one* person?

**SdC:** We are talking now a little bit abstractly, even if quite possibly we are both thinking of concrete projects we have been involved with. There are many different projects but often there will be some key players and maybe these are the people we want to interview. But these key players very often have written down their accounts and when you speak to them, they often just fall back to telling you the things or versions of events they have already put into words. In those cases, you really want to push them to move beyond these well‐rehearsed accounts as these are things you can read up on. Sometimes these can be difficult interviews. In contrast, other people who have kept a bit more in the background for whatever reasons or have been written out of the records, like perhaps technicians, often provide fresher insights.

**LK:** I once found a book that a biomedical researcher was writing about research that he himself was involved in, and in his acknowledgment, he thanked oral historians for doing an interview with him that he then used to write the book.^15^ There it comes full circle.

**SdC:** But, you see, I can even see that, because sometimes in the interview, interviewees see new connections or learn new things. Because they have lived through something, but now looking back and asking these connecting questions, they sometimes see something in a different light.

**LK:** That's a very nice way to put it, because it makes visible the epistemic productivity of the research interviewer, right? It stresses that not everything was clear to the interviewees themselves.^16^

## 7 Reflecting on Historians’ Stances Towards Interviews in Historical Research

**LK:** I think we're nearing our last questions. How do you look at other scholars’ use of research interviews? Does it increase your appreciation of scholarship in contemporary history of science, if the author has made good use of research interviews? And is interviewing something that you advise your graduate students to pursue?

**SdC:** Yes, I'm interested to see how other scholars or historians of science use interviews and especially also how they weave their interviews into their texts. Sometimes they do this in quite interesting ways. And yes, I certainly encourage my students who work on recent historical projects to consider doing interviews. Having to go through the IRB puts a hurdle in the way. But if it's just for a seminar paper, nobody would ask for your IRB.

We have already touched on this, but doing interviews adds a personal dimension to your research, which I really think adds to the fun of it. Because you don't just deal with old administrative documents or something, but you also have the actors’ point of view. This enriches the research experience.

**LK:** That sounds like a beautiful end of the interview. But maybe you still have some questions that you think should have been posed?

**SdC:** I thought this was an excellent set of questions, thank you. One thing that always startles me is that when I mention the interviews to colleagues, they react very strongly to it. They either say something like, “Oh, I'm so glad all my actors are dead.” I have heard this repeatedly. To them it's a frightening idea that their actors might interfere with their work. And others say, “Oh, I wish I could speak to my actors.” Maybe it has to do with the specific actors you have in mind and the role they play in your story. I mean, if you think they did terrible things and you say so clearly in whatever you write, then you don't want them to be able to speak back, right? I don't know how much of it has to do with the kind of actors you have in mind and how pleasant or unpleasant the encounter would be, or whether it has more to do with the attitude towards history. There is a strong feeling among some historians that you need to have historical distance from the people and events you write about. And if the events are too close in time, you can't write the history; it may be sociology or journalism, but it's not history. It's difficult to make out what exactly is at play in these different reactions.

**LK:** I really like that. It echoes many of the other responses that you gave. Maybe we can learn as much about how we think about historians as we can learn about how we think about sources when thinking about the research interviews.

**SdC:** I do think this is true. And that's maybe also a good point to end on. Maybe because interviews are still a contested form of evidence, they make us think harder about the tools and attitudes of historians. That is also the reason why I wrote that article years back as I somehow felt I had to legitimize my use of interviews. There is still resistance to the use of interviews in historical research, even if a little less so to the use of deposited interviews that are often accessible as transcripts and so have acquired more of a text‐like quality. But when you do your own interviews, you have to reflect a little harder on your research practice and the value of your evidence. Of course, other kinds of historical evidence can trigger similar reflections, but when conducting an interview, the historian is more actively engaged in the production of the source.

**LK:** You have to make yourself in a way more visible too.

## 8 Conclusions

**SdC:** I think we can come to the conclusion, one of the conclusions, that it's very difficult to come up with general rules because it really very much depends on the kind of project that is at stake. We can agree on some general guidelines about what it means to do these interviews, what can be gained and what the pitfalls are, but the rest needs to be decided case by case, starting with the question whether it makes sense to embark on a set of research interviews.

**LK:** I was just thinking about an additional conclusion that I think has also come to light. You were saying often the interviewees are cautious when talking about colleagues. And we have also been very cautious in this interview talking about our interviewees. That might be something that is, again, different when you know your actors. You think twice about how you engage with these sources. Because you know that there can be a lot of things involved and that it's also a kind of history that you write about; it won't be neutral to these people.

**SdC:** Yeah. The fact that we know our actors keeps us more accountable.

**LK:** It can also inspire a different approach to sources in general. On a very practical level, it reminds you that the history is also about the people.

**SdC:** Yes, absolutely. Another thing we haven't talked about is to what extent are we writing for a historical audience and to what extent are we writing for scientists? This is an issue for historians of science. I always think our histories are certainly not how scientists would tell the story. But if scientists can't make any sense of the way we speak about the events they have been involved in, then that is a bit problematic. They can agree or disagree with what we write, or they can find it interesting or not interesting, but if they can't relate to it at all, then that is worrisome. If you have talked to the people in the process, I think then you're a little bit more accountable. And this can work in different ways. You can't just use them as sources and then leave them in the cold.

**LK:** Yeah. But it also shows that you are building bridges between history of science and the science that you're looking at.

**SdC:** I think both directions are important and ideally, we would manage to talk to both sides. I have not thought about this before, but in a way, interviews can help, as you say, building some of these bridges. If only by taking whatever scientists have to say seriously.

**LK:** Thank you very much, Soraya! I will now stop the recording.

## Footnotes

1
de Chadarevian 1997; see also Worliczek and Freeborn, this issue.

2
Söderqvist 1997.

3
Benison 1967; Benison 1971.

4
Söderqvist 2003.

5
Thompson 1978.

6 On the history of IRBs and how they have shaped research practices in biomedicine, see Stark 2011.

7
de Chadarevian 2020.

8
Nott 2020.

9 On the debate around this issue in the US context, see White 2017. The source of the confusion around the issue may lie precisely in the difference between life stories and research interviews that is generally not clearly spelled out and of course there are also grey areas between the two kinds of oral history projects.

10 On critical connections between ethnographic field work, including interviews, and history of science, see Dent 2022. On the changing notions of field and fieldwork, see also the other essays in the same *Isis* Focus section: Brinitzer and Benson 2022.

11 For a related point regarding public opinion on animal experimentation, and its impact on the anonymization of research interviews with animal technicians, see Myelnikov, this issue.

12
de Chadarevian 2020.

13 The point on gaining a “feel” for the interviewee draws on Seldon and Pappworth 1983.

14 This is also a focus in this special issue, see Dent and Vimeiro‐Gomes, this issue; Brinitzer, this issue.

15
Tanzi and Parson 2000.

16 Joana Radin makes a related point, when reflecting on how her interviewee developed an awareness of “becoming a historical subject” through their interactions. Radin 2022 on 268.
